# Effectiveness of a myofunctional therapy program in the treatment of mouth breathing in patients with dental malocclusion: a pilot clinical trial

**DOI:** 10.1590/2317-1782/e20250033en

**Published:** 2026-03-30

**Authors:** Letícia Korb da Silva, Giovana Miranda de Brito, Asenate Soares de Matos Pereira, Licia Coceani Paskay, Giédre Berretin-Felix

**Affiliations:** 1 Faculdade de Odontologia de Bauru – FOB, Universidade de São Paulo – USP - Bauru (SP), Brasil.; 2 Programa de pós-graduação em Fonoaudiologia, Faculdade de Odontologia de Bauru – FOB, Universidade de São Paulo – USP - Bauru (SP), Brasil.; 3 Departamento de Fonoaudiologia, Faculdade de Odontologia de Bauru – FOB, Universidade de São Paulo – USP - Bauru (SP), Brasil.; 4 Academy of Applied Myofunctional Sciences – AAMS - Los Angeles (California), USA.; 5 Departamento de Fonoaudiologia, Faculdade de Odontologia de Bauru – FOB, Universidade de São Paulo – USP - Bauru (SP), Brasil.

**Keywords:** Mouth Breathing, Myofunctional Therapy, American Speech-language-hearing Association, Malocclusion, Angle Class II, Respiration

## Abstract

**Purpose:**

To verify the effectiveness of an orofacial myofunctional program aimed at treating respiratory function in adults with dental malocclusion.

**Methods:**

Thirteen adults with Class II malocclusion undergoing orthodontic treatment participated in this pilot study, randomly divided into an experimental group (EG; n=9) and a control group (CG; n=4). Protocols for oral health-related quality of life (OHIP-14) and respiratory symptoms were applied. Respiratory function was assessed using the MBGR protocol. Additionally, maximum phonation time (MPT) of /s/, nasal peak inspiratory flow, and nasal airflow analysis using a metallic plate were measured.

**Results:**

After the intervention, the EG showed statistically significant improvement in respiratory symptoms (p<0.001), MBGR score (p<0.001), respiratory type (p=0.041), respiratory mode (p=0.029), MPT (p=0.002), and nasal peak inspiratory flow (p=0.002). There were no changes in oral health-related quality of life for either group (p>0.05). Three months later, the EG maintained the results, while the CG showed worsening of respiratory symptoms (p=0.003) and MPT (p=0.013).

**Conclusion:**

The orofacial myofunctional therapy program improved respiratory parameters in oral-breathing adults with malocclusion, with no impact on oral health-related quality of life.

## INTRODUCTION

Breathing is a vital function of the organism, developed in the first moment of life, and the nasal cavities have ideal basic conditions to filter particles and microorganisms from the air and allow it to reach the lungs at the ideal temperature and humidity and with good oxygenation^(1-3)^. The impossibility of breathing through the nose leads the individual to develop oral breathing that can have adverse effects on the development of the stomatognathic system (more likely to have learning difficulties than nasal breathers)^(2,4)^ .

Mouth Breathing Syndrome (MBS) occurs when an individual does not breathe efficiently through the nose and starts with the mixed respiratory mode, with the nose being supplemented by the mouth^(5,6)^. It is related to genetic factors, habits or nasal obstruction of varying severity and duration^(3,7)^. Respiratory breathing prolonged is associated with modifications in the craniofacial growth and development pattern, which can result in aesthetic and functional disharmonies ^4, 5, 8.^ Changes that occur in the medium or long term, resulting from MBS, can have harmful consequences for the individual's quality of life due to their personal, physical, psychological and social impact. During adolescence, critical phases of growth, mouth breathing can lead to compensatory adaptations in the orofacial complex, including narrowing of the maxilla, increased anterior facial height, mandibular and occlusal retrognathism and discrepancies. These structural alterations can compromise not only facial aesthetics, but also the functionality of the stomatognathic system, interfering with functions such as mastication, deglutition and phonation^(1-8)^.

Understanding the impact of mouth breathing on craniofacial growth is essential to justify the need for early and effective interventions. Precise identification and adequate management of Mouth breather Syndrome MBScan minimize adverse effects and improve clinical outcomes, optimizing orofacial development and preventing future complications. Thus, MBS is recognized not only as a condition of clinical interest, but also as a relevant public health problem, due to its high prevalence and long-term repercussions^(5)^. Few scientific studies have detailed therapeutic approaches or used objective tests to assess the efficacy of orofacial myofunctional therapy on respiratory function. One study proposed a short-term treatment for children and adolescents with orthodontic issues, focusing on improving breathing, speech, harmful oral habits, eating, oral hygiene, and posture^(9)^. Previous research evaluated the influence of myofunctional therapy on the oral muscles and lip posture of children who mouth breathe without nasal obstruction^(10)^. Another study assessed the effectiveness of removing sucking habits combined with orofacial myofunctional therapy in expanding nasal ventilation in children^(11,12)^ studied speech therapy in patients with asthma, allergic rhinitis, and mouth breathing, concluding a significant impact on respiratory function. However, the research primarily focused on children and adolescents, not addressing adults.

In addition, to date, few studies have been published involving evaluation of the upper and lower airways in individuals undergoing orthodontic treatment^(13,14)^, and no studies were found that applied myofunctional therapy for the treatment of habitual mouth breathing. In adults, however, the literature indicates a high occurrence of mouth breathing in individuals with malocclusion. Therefore, the objective of this study is to verify the effectiveness of an orofacial myofunctional program aimed at the treatment of habitual mouth breathing in adults with dental malocclusion.

It is expected that this course of action will make it possible to change and optimize the breathing mode presented by patients even in the presence of malocclusion, bringing benefits to the quality of life, as well as more favorable dento-occlusal results at the conclusion of the dental treatment.

## METHODS

This pilot clinical trial screened a total of 254 young adults with dental malocclusion who were undergoing orthodontic treatment at the Orthodontics Clinic of Bauru School of Dentistry – University of São Paulo, and other orthodontic clinics in city of Bauru, SP, Brazil with the agreement of their administrative directors. This study was conducted only after approval by the institution’s Research Ethics Committee (approval number 1,198,937). After the clinical inclusion and exclusion criteria were applied, 35 patients were eligible to start the therapy program. However, after applying the instrumental criteria, 13 patients remained eligible for the start of the intervention. Of these, 13 patients expressed interest and availability to participate in the study and were allocated to the experimental and control groups.

The determination of the sample size was based on a power analysis of the statistical test, with a significance level of 5% and a test power of 80%, considering a medium effect size for the proposed intervention. The calculation indicated the need for eight participants in the experimental group and five in the control group to ensure an adequate evaluation of the effectiveness of the therapy program.

**Figure 1:**
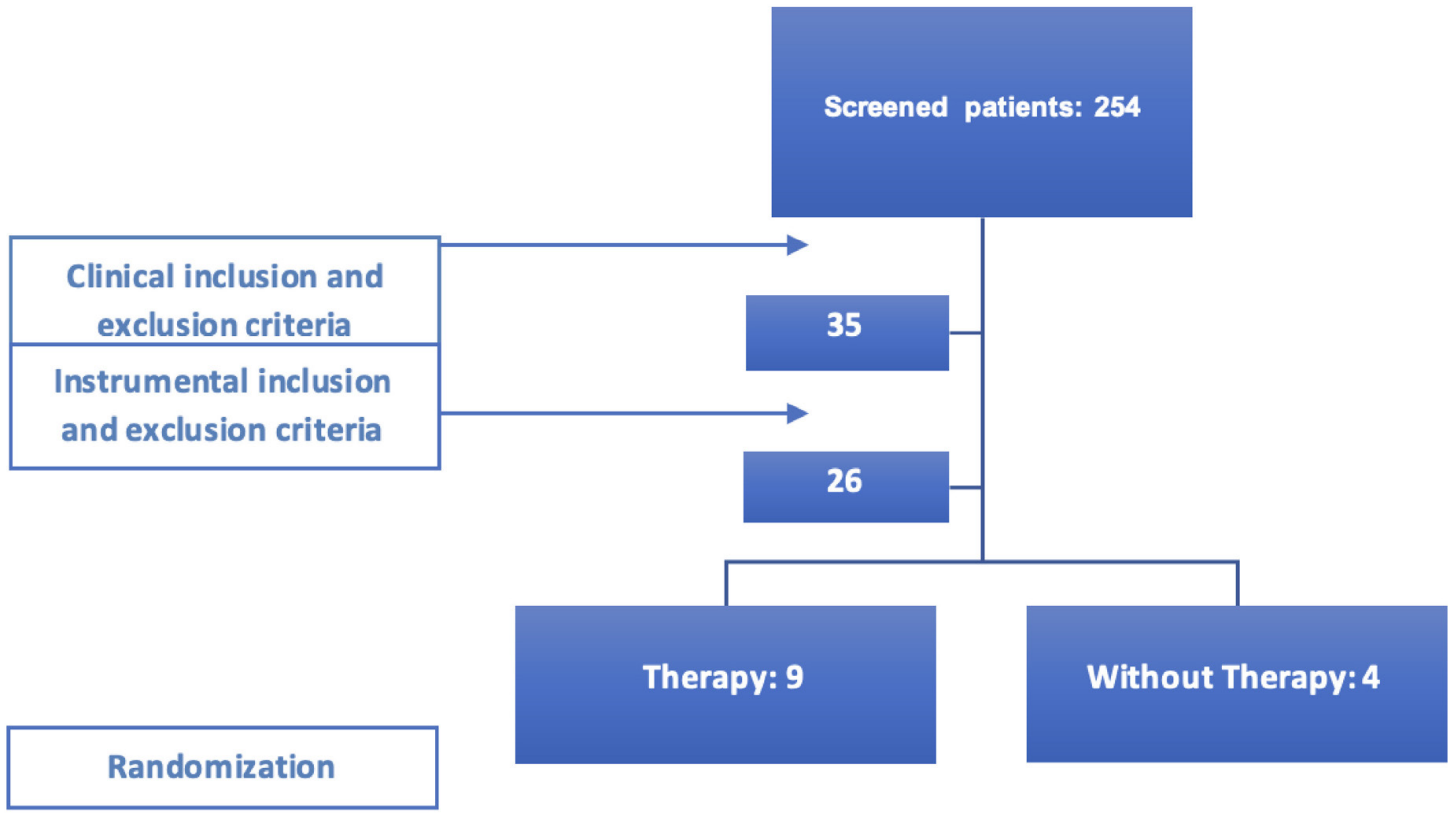
Flowchart of patient inclusion and distribution, according to inclusion and exclusion criteria, patient availability and randomization

The 13 participants were randomly distributed into two groups: experimental group (n = 9), submitted to the Therapy Program, and control group (n = 4), who did not receive intervention during the study period (Figure 1). Randomization was performed using a simple process in Microsoft Excel software, using the RAND function to generate a random sequence of numbers attributed to the participants. The allocation of subjects was done by an independent researcher, ensuring blinding in the distribution of the groups.

Patients with a previous diagnosis of severe Class II dental malocclusion, identified by the orthodontist responsible for the clinical evaluation and classification, undergoing orthodontic treatment, in addition to presenting respiratory oral and oronasal diagnosis documented as non-obstructive by means of a speech-language pathology and otorhinolaryngological evaluation, were included in the study. The exclusion criteria included: mouth breathing habit, severe posterior crossbite, craniofacial syndromes, history of tabagism, history of chronic pulmonary diseases.

The research was approved by the Research Ethics Committee and the approval number of the Research Ethics Committee. All individuals involved signed the Free and Informed Consent Form.

## PROCEDURES

For the study group, such assessments were performed one week before starting therapy, one week after the end of therapy and three months after the application of the Orofacial Myofunctional Therapy Program, counting from the last day of the session (Orofacial Myofunctional Clinical Assessment, Peak Inspiratory Flow Assessment, Measurement of Maximum Phonation Time and Assessment of Oral Health-Related Quality of Life and Respiratory Symptoms). For the control group, an initial assessment was performed, and the exams were repeated three months after the first assessment. The assessments were conducted by a speech-language therapist with clinical and research experience, who was not informed about the group to which the patient belonged.

Intra-examiner reliability was verified using the intraclass correlation coefficient (ICC), with values above 0.90, indicating excellent agreement. For the ICC calculation, data from 20% of the sample were used, with reassessments conducted at a 15-day interval. The reliability analysis was specifically applied to the “breathing” item of the MBGR protocol (including respiratory type, respiratory mode, possibility of nasal use, and expiratory airflow). ICC values refer to the scores obtained in these domains, analyzed both separately and in aggregate. All assessments were conducted by a speech-language pathologist blinded to the allocation of participants in the experimental and control groups.

## OROFACIAL MYOFUNCTIONAL CLINICAL ASSESSMENT

### Assessment of respiratory functions

For the clinical assessment of breathing, all items covered for this aspect in the MBGR Orofacial Motricity Protocol were analyzed, considering the possibilities of responses and the scores assigned by the authors^(15)^. As for the respiration type, this was evaluated by observing the individual at rest sitting and standing, and by palpating the thoracic and abdominal regions. Regarding the breathing mode, it was evaluated by observing the individual at rest for 1 minute, by verifying the position of the lips, jaw and tongue, by noting the presence of some sealing point in the oral cavity, and by breathing being classified as nasal, oronasal or oral. To verify the possibility of using the nasal route for breathing, individuals were asked to keep a little water in their mouths for 2 minutes. The expiratory airflow was also observed using the Altmann millimetered nasal mirror (Pró-Fono), positioned under the participants' nostrils.

### The expiratory airflow

Nasal aeration was assessed using the Altmann millimetered nasal mirror (Pró-Fono), positioned below the nostrils to capture the condensation area during forced nasal expiration. For the procedure, the participant remained seated in an orthostatic position, breathing normally. Then, they were instructed to exhale forcefully through the nose, keeping their mouth closed, directing the expiratory flow towards the mirror's surface. The examination was repeated three times, and the condensation area formed on the mirror was measured in millimeters, with the average of the three obtained values being considered for analysis.

### Peak inspiration flow assessment

The peak nasal flow was evaluated during exclusively forced nasal inspiration, in liters per minute, using the In-Check Nasal® equipment (Clement Clarke International). For the examination, the individual remained seated in an orthostatic position, breathing normally, while the nasal cavity was sealed by means of a mask attached to the nasal flow measuring device. The patient was instructed to perform three breathing cycles in the usual way and then to inhale as deeply and strongly as possible through the nose, keeping the mouth closed. The values of three repeated measurements were recorded to calculate the mean.

### Maximum phonation time measurement

The available air support, closely related to the breathing pattern, was evaluated by asking the patient to make the hissing sound /s/, measuring the time of the sound recorded on a stopwatch, only once, if the patient demonstrated understanding of the instructions and the exam result was consistent.

Assessment of quality of life in oral health and respiratory symptoms

The quality of life (QL) assessment questionnaire was applied to oral health by using the Oral Health Impact Profile simplified - OHIP-14^(16)^. This protocol contains 14 questions distributed into seven categories that make up the questionnaire and contains two questions related to “problems with the teeth”. In the present study, the questions were related to orthodontic alterations. The total score obtained corresponded to the sum of the scores obtained from each of the questions, with 56 points being the maximum individual response: the higher the score, the worse the QL.

The subjects also answered a questionnaire on respiratory symptoms proposed by Caouette-Laberge et al.^(17)^ and translated and adapted by Yamashita and Trindade^(18)^. This questionnaire addresses issues such as respiratory obstruction, breathing problems, shortness of breath, drowsiness, or loss of smell. To obtain a score, an adaptation of the questionnaire was made, as follows: for “yes” answers, a score of 1 was assigned. For “no” answers, a score of 2 was assigned, since the lower the score, the worse the complaints of respiratory symptoms reported by the patient.

Orofacial myofunctional therapy program applied to respiratory function:

In the selected individuals with oral or oronasal breathing, a therapy program was applied by the researcher in charge, in order to optimize the nasal respiratory function. The program presented in Chart 1 was based on the proposals previously described in the literature review of this project, which demonstrated effectiveness in the therapy results. In addition, it addressed the use of breathing for speech, as this aspect proved to be impaired based on results obtained in a previously developed study^(14).^

**Chart 1:** Therapy Program proposed for the treatment of Mouth Breathing

**Objective**	**Means to achieve goals**	**Description**
1. To rearrange overall body posture	Carrying out an exercise to stretch the cervical muscles*.	- Instruct patients to perform pendulum movements with their head four times, starting twice from right to left, and twice from left to right.
	Exercise to stretch the lateral spine muscles *	- Instruct patients to stretch the arms up and lower the trunk towards the feet. Perform three times, keeping their arms extended for 15 seconds.
	Awareness and modification of body posture**.	- Ask patients to, with their eyes closed, perceive their body in relation to the posture.
		- Ask patients to sit in front of a mirror and perform self-monitoring to modify and maintain their posture with the shoulders and head aligned, forming 90º angles between the trunk and hips, knees and feet.
2. To raise awareness of respiratory function.	Perception of the performed breathing pattern^*^	- Ask patients to perform self-monitoring in relation to their breathing pattern, perceiving the use or not of the mouth as a way of breathing, as well as the movement of the diaphragm and thoracic regions during function.
	Awareness of normal/adequate physiological pattern**.	- Show patients, through videos and images, the ideal breathing pattern.
3. To perform nasal cleaning.	Application of saline solution in the nostrils^**^.	- Insert 5 ml of saline into the patients’ nostrils, with the help of a syringe. Patients should have their head flexed backwards. Ask them to force the introduction of the liquid into their nasal cavity. After cleaning, ask them to blow their nose, alternating nostrils.
		- Perform only one cleaning in each nostril, as well as the process of blowing their nose.
4. To promote use of the nasal airway.	Performing nasal inhalation and exhalation exercises**.	- Ask patients to inhale air only through one nostril (occluding the other with a finger) and exhale the air through the previously occluded nostril, closing, with the finger, the nostril that was used to inhale the air. Perform it five times.
5. To strengthen the muscles: orbicularis oris, buccinator, jaw elevators, intrinsic and extrinsic of the tongue.	Performing orofacial myofunctional exercises**.	- Perform counter-resistance exercises with a spatula in the three regions of the orbicularis oris of the lower and upper lips. Hold the spatula making a movement contrary to the muscle for 10 seconds, for three series.
		- Perform counter-resistance exercises with a spatula in the middle bundle of the buccinator muscle, for 10 seconds, for three series.
		- Carry out resistance exercises of the tongue against the palate, for 10 seconds, for three series.
		- Perform mouth opening and closing exercises keeping the apex of the tongue in the region of the incisive papilla (three series of 10 movements). Then, perform digital counter-resistance against the lower premolars for 10 seconds, for three sets.
6. To establish the nasal airway at rest.	Nasal airway training**.	- Ask patients to breathe only through the nose, initially, for 2 minutes, keeping the mandible elevated and anterior oral sealing (through lip occlusion or contact of some region of the tongue against the palate). During this time, patients read a text silently or watch a video. One minute per session should be added to the nasal breathing maintenance time, until reaching 5 minutes.
7. To promote lower middle respiratory type.	Respiratory and pneumophonoarticulatory training involving the emission of phones*.	- Request inspiration and expiration while counting numbers, performed by the therapist in a rhythmic way. Each number counted corresponds to one second.
		Training starts inspiration (nasal mode and lower middle type) at 4 seconds and expiration at 6; increasing in the next session by 6 seconds to the inhalation and exhalation by 9. The goal of this workout is to inhale in 7 seconds and exhale in 14 seconds.
		- Ask patients to breathe in air (nasal mode and lower middle type) and perform oral expiration, without using reserve air. Perform it three times.
		- Ask patients to breathe in air (nasal mode and lower middle type) and perform the sustained emission of the phoneme [s], without using reserve air. Perform it three times
		- Ask patients to breathe in air (nasal mode and lower middle type) and perform the sustained emission of the phoneme [z], without using reserve air. Perform it three times.
		- Ask patients to breathe in air (nasal mode and lower middle type) and perform the emission with the passage of loudness from the phone [s] and to the phone [z], without using reserve air. Perform it three times.
		To monitor the increase in maximum phonation time, a stopwatch is used. As a goal, the normality time for adults is taken into account. For /s/, 15 to 25 seconds. For the other phonemes, 16 to 18 seconds.
8. To promote pneumophonoarticulatory coordination.	Training of pneumophonoarticulatory coordination involving the reading of sentences and texts*.	- Ask patients to read short, medium and long sentences.
		Ex.: Inhale- THIS IS JOHN- Exhale.
		Ex.: Inhale- THIS IS JOÃO'S HOUSE- Exhale.
		Ex.: Inspire - THIS IS THE GRAIN THAT WAS IN JOÃO'S HOUSE - Exhale. (PICOLOTTO, 1996).
		- Ask patients to read a text, making the necessary pauses for inspiration, taking care not to use reserve air to avoid hyperfunction of the vocal muscles. Every time this exercise is carried out, a text different from the one proposed in another session is selected.
9. To promote nasal breathing during masticatory function.	Respiratory training during chewing**.	- Offer patients their favorite cookie and request that chewing be performed using only the nasal cavity for breathing. Three portions of 2 cm each are offered.
10. To promote coordination between breathing and swallowing functions.	Respiratory training during the swallowing of solid and liquid foods**.	- Ask patients to place a portion of food in their mouth while breathing through their nose, prepare the food for swallowing, pause in breathing, swallow the food and exhale after swallowing. Three portions of cookies, 2 cm each, are offered; 10 portions of 15 ml of water, fractionated into 5 ml by swallowing.

* The patient performed the activity in a standing position;

** The patient performed the activity in a sitting position.

A total of twelve therapy sessions were proposed, each lasting 45 minutes and conducted twice a week. Participants also received a therapeutic handout with detailed instructions for home-based exercises. The prescribed routine included daily nasal irrigation with saline solution (once a day), nasal breathing training alternating nostrils (10 repetitions), lip mobility exercises (10 repetitions), tongue mobility exercises (sweeping the palate – 10 repetitions), a tongue strength exercise (pressing the tongue tip against the palate for 10 seconds), and maintaining appropriate tongue posture at rest. These exercises were to be performed three times daily. Adherence to the home program was monitored through participants’ self-reports and clinical performance during follow-up sessions.

### Statistical analysis

To compare the quantitative data of the same group between the pre- and post-therapy periods, the paired t-test was used for the following variables: OHIP-14, respiratory symptoms protocol, respiratory function from the MBGR protocol, maximum phonation time, inspiratory nasal airflow, and expiratory nasal airflow. For qualitative data from the same group at different phases, the McNemar´s test was applied to the following variables: respiratory type, respiratory mode, expiratory nasal airflow, and possibility of nasal breathing. For comparisons between groups, Fisher's Exact Test and the Chi-Square Test were used. The software used was Jamovi, version 2.3.26.0.i. A significance level of 5% (p<0.05) was adopted.

## RESULTS

To enable comparisons between the pre-and post-immediate periods of treatment and the untreated group in the pre-and post-three months period, the OHIP-14 and Respiratory Symptoms questionnaires were applied. In addition, the Orofacial Myofunctional Assessment was performed, through the application of the MBGR protocol, considering the Respiratory Function regarding the respiratory type, breathing mode, nasal flow, and possibility of nasal use.

To obtain quantitative instrumental data, the Maximum Phonation Times and Maximum Nasal Inspiratory Peak (Peak-Flow) exams were performed. Additionally, the measurement of the area of the expiratory nasal airflow was calculated.

The results (OHIP-14 and Respiratory Symptoms; Orofacial Myofunctional Assessment; Maximum Phonation Times and Maximum Nasal Inspiratory Peak) of each group are presented in the pre, immediately post and 3 months post therapy, as well as the comparison between the experimental and the control groups.

### Quality of life in oral health

When considering the total score, a mild to moderate impact was found, at both times and for all patients evaluated. The most scored domains for patients in the experimental group were functional limitation, physical pain and psychological discomfort. As for the control group, it was possible to observe the physical limitation, in addition to the domains mentioned for the experimental group.

After the orofacial myofunctional treatment, there was still a recurrence in responses related to functional limitation, physical pain, psychological discomfort, physical limitation, psychological limitation, social limitation and disability in both groups, suggesting no improvement of quality of life in oral health for patients treated with myofunctional therapy. The individual results related to quality of life in oral health, investigated through the application of the OHIP-14 protocol, in the pre- and post-intervention periods, are presented in Table 1.

**Table 1:** Distribution of results according to the domains of the OHIP-14 quality of life questionnaire and the total score, in the pre-therapy and immediate post-therapy periods, for the experimental (EG) and control (CG) groups

Group	Patient	**Domains**														**Total Score**	
		**Functional limitation**		**Physical pain**		**psychological discomfort**		**physical limitation**		**psychological limitation**		**social limitation**		**Inability**			
		Pre	Post	Pre	Post	Pre	Post	Pre	Post	Pre	Post	Pre	Post	Pre	Post	Pre	Post
EG	1	1	3	2	5	1	4	0	0	3	2	0	2	0	0	7	16
	2	0	1	0	2	0	0	0	0	0	0	0	0	0	0	0	3
	3	0	0	3	1	0	0	0	0	0	0	0	0	0	0	3	1
	4	0	0	0	0	0	0	0	0	0	0	0	0	0	0	0	0
	5	1	0	2	0	1	0	0	0	0	0	0	0	0	0	4	0
	6	0	0	2	2	0	0	2	1	0	0	1	1	0	0	5	4
	7	3	2	4	5	0	2	0	0	0	0	0	0	0	0	7	9
	8	2	0	0	8	8	2	6	5	4	4	1	0	2	0	23	19
	9	0	0	0	1	0	2	0	1	2	4	2	2	0	0	4	10
CG	1	2	3	2	2	0	0	0	1	0	0	2	2	0	0	6	8
	2	1	2	3	2	0	1	2	1	0	0	0	0	0	0	6	6
	3	3	2	1	1	0	0	1	1	0	0	2	2	0	0	7	6
	4	4	4	3	4	3	4	2	2	0	1	2	2	0	0	14	17

**Caption:**Table 1 presents the domain scores of the OHIP-14 questionnaire in the pre- and post-therapy periods for the experimental (EG) and control (CG) groups. The scores range from 0 to 4, as follows: 0 = never, 1 = almost never, 2 = sometimes, 3 = almost always, and 4 = always. The total score corresponds to the sum of the scores from the seven assessed domains. The table allows for a direct comparison between the evaluation moments, highlighting the individual variation of each participant

### Respiratory symptoms

The respiratory symptoms questionnaire showed that the patients' responses varied, and all reported breathing through the mouth and having difficulty breathing during some physical activity, with similarity between the groups in the pre-therapy period.

The paired t test was performed, with a significant difference in the value of p<0.001 for the experimental group (EG) and p=0.003 for the control group (CG) , and when comparing the results obtained in the different periods (pre and post-therapy). There was an improvement in the complaints reported by patients in the experimental group. As for the control group, there was a worsening of complaints and symptoms.

The improvement found for the experimental group was maintained after 3 months of therapy, while the worsening of the scores obtained for patients in the control group, found in the post-immediate period, remained at the 3-month follow-up (p>0.05).

### Orofacial myofunctional assessment of respiratory function

All patients evaluated had medium/upper respiratory type. The nine individuals who completed the therapy program started to present medium/lower respiratory type and nasal breathing mode. In addition, the breathing mode of six patients was oronasal and one was oral. Table 2 shows a statistically significant improvement in the EG, according to the MBGR Protocol score, since after the therapy period they presented lower score values. The control group, on the other hand, showed no changes in their scores with regard to the assessment of respiratory function in the MBGR Protocol.

**Table 2:** Comparison between the experimental and control groups in the pre- and immediate post-therapy periods regarding quality of life in oral health (OHIP-14), respiratory symptoms, orofacial myofunctional assessment of respiratory function (MBGR), maximum phonation time (MPT), and peak nasal inspiratory flow

OHIP-14	CG	EG	P value
	Mean ± SD	Mean ± SD	
		
Pre	8,25 ± 3,86	6,11 ± 6,90	0.579
Post	9,25 ± 5,25	5,42 ± 6,42	0.337
**Respiratory Symptoms**			
Pre	31,25 ± 3,40	29,00 ± 4,35	0.383
Post	29,00 ± 3,16	34,44 ± 4,33	0.047*
**MBGR**			
Pre	4,25 ± 0,957	3,78 ± 1,20	0.505
Post	4,25 ± 0,957	1,00 ± 1,11	0.000^*^
**MPT**			
Pre	12,31 ± 0,923	10,35 ± 4,38	0.406
Post	10,95 ± 1,13	17,61 ± 4,12	0.010*
**Inspiratory Peak**			
Pre	57,50 ± 8,76	61,89 ± 10,22	0.474
Post	54,58 ± 7,12	93,15 ± 15,57	0.001^*^

Statistics: t test

P-values marked with an asterisk (*) indicate statistically significant differences (p < 0.05)

**Caption:**Table 2 presents p-values and standard deviations (SD) obtained from the comparison between the pre- and immediate post-therapy periods in the experimental (EG) and control (CG) groups, related to the following outcomes: oral health-related quality of life (OHIP-14), respiratory symptoms, respiratory function assessed by the MBGR protocol, maximum phonation time (MPT), and peak nasal inspiratory flow. The paired t-test was used for statistical analysis

Using the paired t-test, it was possible to observe that, in the respiratory function assessment, using the MBGR protocol, the EG showed a significant reduction in mean scores from the pre- to post-therapy period, with a mean of 3.78 (SD = 1.20) before the intervention, and 1.00 (SD = 1.11), after the intervention (p < 0.001). In contrast, the CG maintained similar mean values across both periods, with a mean of 4.25 (SD = 0.95), showing no statistically significant difference (p = 1.0).

### Respiratory Type **(**MBGR Protocol)

Regarding the respiratory type, it was possible to observe that, of the nine patients treated, six (66.7%) started to present a lower medium type, resulting in a statistically significant improvement. No changes were observed in the control group.

### Breathing Mode **(**MBGR Protocol)

With regard to the breathing mode, it was observed that only 11.1% of the treated patients did not change this parameter while 88.9% of these patients showed a significant change (p<0.05) by becoming nasal breathers. The control group showed no changes in terms of their breathing mode (p=1,000).

### Nasal expiratory flow **(**MBGR Protocol)

Although a change was found in the pattern of expiratory flow, in which three patients (33.3%) no longer had reduced flow bilaterally, there was no statistically significant difference when comparing the pre- and post-therapy periods for the experimental group. However, it was noted that the aspects of the respiratory flow in the control group remained unchanged at the 3- month follow-up.

### Possibility of nasal use **(**MBGR Protocol)

In the post-therapy period, five (55.6%) of the patients in the experimental group showed to have changed the possibility of nasal use for more than 2 minutes, which is expected to be the normal pattern in non-obstructive cases. When evaluating patients at the completion of 3 months of treatment, it was possible to observe that these parameters remained adequate in 100% of treated patients. However, no statistically significant difference was observed for this aspect. In the control group, on the other hand, this breathing aspect remained unchanged, when comparing the pre and post 3-month periods.

### Maximum phonation time

The EG demonstrated a statistically significant improvement in Maximum Phonation Time (MPT) after the therapeutic intervention (p = 0.002). The average MPT increased by 10.36 seconds (±4.39) in the pre-therapy period to 17.62 seconds (±4.13) in the post-therapy period. The highest values were 23.97 s, 22.88 s, and 21.98 s, evidencing a positive response to the intervention, although only part of the subjects reached values comparable with normality standards for adults (>20 s).

In contrast, the (CG), which did not receive intervention, showed a significant reduction in MPT, with the average decreasing from 12.32 seconds (±0.92) to 10.96 seconds (±1.14) (p = 0.013). No participant in the control group reached adequate standards in the post-test, and all presented a worsening of their phonatory values over time.

### Peak of nasal inspiratory flow

According to^(19)^, the average peak inspiration for healthy patients is 102.91 liters/minute. In this context, it was possible to verify that the values obtained for all evaluated patients were altered. When comparing the results obtained for the pre- and post-intervention periods, treated individuals showed an increase in nasal inspiratory peak, with 33.3% of these reaching normality, which was not found in the untreated group. While performing the evaluation after 3 months of treatment, it was possible to observe that the modified standards were maintained, with regard to the comparison between immediate post and 3 months post, showing no statistically significant difference.

### Expiratory nasal airflow area

The expiratory nasal airflow areas, measured in millimeters on the millimetered metallic mirror, showed discrete variations between the pre- and post-evaluation periods in both groups. In the experimental group (EG), the average obtained measurements were 22.63 mm at the pre-therapy moment and 21.79 mm at the post-therapy moment (p = 0.064). In the (CG), which was not submitted to the intervention, the average measurements were 16.82 mm at the pre-evaluation and 17.18 mm at the post-evaluation (p = 0.972).

The statistical analysis indicated that there was no statistically significant increase in the mean nasal expiratory airflow in either of the evaluated groups, making it important to highlight that the control group did not undergo any type of therapy between the two collection moments.

Therefore, as presented in Table 2, the experimental and control groups were similar before the application of the myofunctional therapy program. After the therapeutic intervention, the groups became different in relation to Respiratory Symptoms, the total score of the respiratory function of the MBGR Protocol, Maximum Phonation Time, and Peak Nasal Inspiratory Flow.

## DISCUSSION

Due to the lack of clinical studies on the treatment of oral breathing in adults with dental malocclusion, this pilot clinical trial applied a myofunctional therapy program to address oral breathing in this population.

For the implementation of the therapy program, five clinical studies were selected^(9-12,20)^ with the approach aimed at children, but it was possible to include in the program the objectives and orofacial myofunctional strategies presented in these studies, although adapted for adult individuals. Additionally, nasal breathing training during mastication, coordination between breathing and swallowing function, as well as between breathing and speech, was proposed. The aspects that involve the other orofacial functions in the treatment of mouth breathing are unprecedented, and it is important to adjust the breathing pattern during the performance of the other functions, since these are compromised in patients with mouth breathing.

In this study, the participants' oral health-related quality of life was assessed using the Oral Health Impact Profile OHIP-14^(16)^, widely used to measure the impact of oral health on quality of life. After applying the myofunctional orofacial therapy program for respiratory function, no improvement was observed in the OHIP-14 aspects. One possible explanation is that the protocol did not specifically address respiratory issues, and the patients had not yet completed orthodontic treatment. Göranson et al.^(21)^ showed that individuals undergoing orthodontic treatment experienced a negative impact on oral health-related quality of life, similar to that found in the present study.

During the pre-therapy period, the experimental and the control groups were similar in terms of the Respiratory Symptoms Protocol score. The most frequent symptoms presented were mouth breathing, drowsiness and constant tiredness, and these characteristics have already been observed in another study^(22)^.

After the treatment, the groups showed significant differences in respiratory symptoms, with the treated group showing a lower score (p<0.001) and the control group a worse score (p<0.001). This pattern was maintained three months after therapy for the treated group. The respiratory symptoms questionnaire, used in patients with pulmonary diseases and cleft lip and palate^(23,24)^, proved effective in assessing respiratory complaints and may be a good control indicator for respiratory therapies, as observed in the study.

The MBGR Orofacial Myofunctional Assessment Protocol is commonly applied in research with children^(25,26)^, but it has been validated for application in adults with temporomandibular disorders^(19)^ and used in adults undergoing orthodontic treatment^(27)^.

With regard to the respiratory function score of the MBGR Protocol, it was possible to observe a statistically significant improvement (p<0.001) of the treated patients, since they presented lower score values after the therapy period.

It can be concluded that the therapy program, which addresses the respiratory function aspects of the MBGR protocol, demonstrated sensitivity in assessing and controlling therapeutic effectiveness. Other studies on myofunctional therapy for mouth-breathing patients did not use clinical protocols, making comparison with this research difficult.

After the application of the therapy program, it was possible to observe a change in the respiratory type in 66.7% of the treated patients, and in the period after 3 months of therapy, the results for the respiratory type remained similar to those in the post-immediate period. In a study whose sample consisted of adults with skeletal malocclusion, a predominance of the upper-middle respiratory pattern was found, making professional intervention necessary to promote better lung use and harmonic chest expansion^(14)^. In the present study, the same characterization can be observed in young adult patients with dental malocclusion justifying the need to consider this aspect in the myofunctional therapy.

After applying the therapeutic program, 88.9% of oral or oronasal breathers started to breathe exclusively through the nose, and after three months, 100% showed habitual nasal breathing. The effectiveness of the therapy in adjusting the respiratory mode in individuals without nasal obstructions was confirmed, aligning with previous studies on the treatment of oral breathing in children and adolescents^(11,20)^, which also demonstrated the benefits of speech therapy in modifying the respiratory pattern.

In a published study^(14)^, it was possible to observe that patients with skeletal malocclusion present unilaterally reduced nasal flow. In the present study, we found a similar number of patients with reduced nasal flow uni and bilaterally, despite the participants in the present study having dental malocclusion. No studies with similar casuistry were found so that the findings of the present study could be compared.

Following the therapeutic intervention, 33.3% of the participants who initially presented with bilateral reduction in nasal airflow demonstrated a shift to unilateral reduction. This outcome was sustained at the three-month follow-up. Although the change did not reach statistical significance, it is consistent with previous findings that report improvements in nasal airflow after myofunctional therapy and nasal cleansing procedures^(11)^. It is worth noting that the participants in the present study were classified as habitual mouth breathers; however, they exhibited adequate nasal patency at baseline. This may suggest that nasal airflow was not significantly compromised prior to the intervention, potentially limiting the sensitivity of this measure in detecting subtle post-treatment improvements.

In this study, the "water test"^(28)^ was used to check for nasal obstruction or oral breathing due to habit. If the breathing mode is oral despite the patient being able to use the nose for more than two minutes, it is likely a habit; if less than a minute, there may be a nasal obstruction. Before therapy, five patients showed unexpected changes in the test, indicating that oral breathing habits can impair nasal function, even without obstructions. Although the statistical analysis did not show a significant change, all patients improved their nasal breathing control by keeping their lips sealed during the two-minute test.

Although the literature on orofacial myofunctional therapy in adults remains limited, the findings of the present study reveal important parallels with research conducted in pediatric populations. In the study by Gallo and Campiotto^(20)^, which involved children with oral breathing, ten sessions of therapy focused on strengthening orofacial muscles and training nasal breathing resulted in improvements in lip sealing and a transition to nasal breathing patterns in 66.6% of participants—similar to the present study, in which 88.9% of adult patients shifted to a nasal breathing mode, with maintenance of results at the three-month follow-up. Similarly, Saccomanno et al.^(29)^ emphasized the relevance of myofunctional therapy as an essential tool for reorganizing oronasal functions across all ages, especially when applied through a structured and multidisciplinary approach. Despite anatomical and developmental differences between age groups, the current data suggest that the benefits observed in childhood—such as improved muscle coordination, respiratory patterns, and functional stability—also extend to adults. These findings reinforce the clinical applicability of pediatric protocols, such as the MBGR, to adult populations, provided they are adapted to functional maturity and case severity. Therefore, this study contributes to filling a gap in the existing literature and highlights the need to further expand research involving adult populations.

The experimental group patients showed a significant increase in Maximum Phonation Time (MPT) after the therapeutic program, and this improvement was maintained three months after the treatment ended. The persistent improvement may be attributed to the program's focus on increasing expiratory time and training the middle/inferior respiratory muscles, which enhances respiratory support for speech. In contrast, the untreated group showed a reduction in MPT, possibly due to the persistence of respiratory alterations, negatively impacting other functions dependent on the respiratory system, such as speech support.

For the measurement of the expiratory nasal area, no difference was found between the groups in the pre- and post-therapy periods, both immediately and after 3 months, which may be related to the fact that participants had no obstructive problems, such as deviated septum and increase of palatine and pharyngeal tonsils, or even with possible cases of allergic rhinitis, based on the exclusion criteria adopted for the selection of the sample of this research study.

After applying the myofunctional therapy program, a significant improvement was observed in the patients' respiratory condition, including respiratory symptoms, respiratory function, maximum phonation time, and inspiratory peak. In contrast, individuals who did not adhere to the treatment continued to exhibit altered functional and symptomatic aspects.

This pilot clinical trial demonstrated the effectiveness of the therapeutic program, which was sustained after three months, suggesting benefits for orthodontic treatments by reducing the risk of recurrence. Simultaneously addressing all functions - breathing, chewing, swallowing, and speaking - is crucial.

Despite the promising findings, this study presents some limitations that should be considered for a balanced interpretation of the results and for the improvement of future research. The main limitations are the relatively small sample size and the nature of the pilot study. Future studies should include a larger number of subjects and a more extensive sample. Furthermore, the follow-up period was restricted to three months after therapy, which does not allow for evaluating the durability of the long-term effects. Although the benefits seem to have been maintained during this period, future studies should consider a longer follow-up to determine if the improvements are sustainably maintained over the years, especially in the context of orthodontic treatment.

Another aspect that deserves attention is the potential influence of the exercises performed at home. Participants received therapeutic plans to complement the therapy, but adherence was not systematically monitored. This could have impacted the results in a variable way among the subjects. Future research should incorporate methods for tracking adherence, such as self-report records, caregiver reports, or digital monitoring tools. Additionally, the absence of a sham therapy group limits the capacity to differentiate the specific effects of myofunctional therapy from non-specific factors, such as greater clinical attention or placebo responses. The inclusion of a control group that receives an alternative intervention would help to isolate the real benefits of the proposed therapy.

Given these limitations, the need for studies with larger samples, extended longitudinal follow-up, and more rigorous methodologies is reinforced to confirm the findings and ensure their clinical applicability. Future research with a larger participant pool and longitudinal follow-up is essential to evaluate the durability of therapeutic benefits, the impact of myofunctional therapy on orthodontic treatment outcomes, and the necessity of therapeutic intervention pre- and post-orthodontic appliance removal.

## CONCLUSION

The application of the myofunctional therapy program for the treatment of mouth breathing, in individuals with dental malocclusion, resulted in an improvement in the respiratory parameters with regard to the type and mode of breathing, as well as in the inspiratory peak, increase in the maximum phonation time and decrease in respiratory symptom, however, no impact was observed on oral health-related quality of life.
